# Differential effect of caffeine intake in subjects with genetic susceptibility to Parkinson’s Disease

**DOI:** 10.1038/srep15492

**Published:** 2015-11-02

**Authors:** Prakash M. Kumar, Swe Swe Thet Paing, HuiHua Li, R. Pavanni, Y. Yuen, Y. Zhao, Eng King Tan

**Affiliations:** 1 Duke-NUS Graduate Medical School, Singapore, 8 College Road, Singapore 169857; 2 Department of Neurology, National Neuroscience Institute, 11 Jalan Tan Tock Seng, Singapore 308433; 3 Health Screening Unit, Singapore General Hospital, Outram Road, Singapore 169608; 4 Health Services Research, Singapore General Hospital, Outram Road, Singapore 169608

## Abstract

We examined if caffeine intake has a differential effect in subjects with high and low genetic susceptibility to Parkinson’s disease (PD), a common neurodegenerative disorder. A case control study involving 812 subjects consisting of PD and healthy controls were conducted. Caffeine intake assessed by a validated questionnaire and genotyping of PD gene risk variant (*LRRK2* R1628P) was carried out. Compared to caffeine takers with the wild-type genotype (low genetic susceptibility), non-caffeine takers with R1628P variant (high genetic susceptibility) had a 15 times increased risk of developing PD (OR = 15.4, 95% CI = (1.94, 122), P = 0.01), whereas caffeine takers with R1628P (intermediate susceptibility) had a 3 times risk (OR = 3.07, 95% CI = (2.02, 4.66), P < 0.001). Caffeine intake would significantly reduce the risk of PD much more in those with high genetic susceptibility compared to those with low genetic susceptibility.

Parkinson’s disease (PD) is a common neurodegenerative disorder, is characterized clinically by tremor, muscle rigidity, bradykinesia and postural instability. Both genetic and environmental factors have been associated with the pathogenesis of PD and their role in the etiology of PD has been debated. The relative contribution of genetic and environmental factors and their interactions is still unclear^1^.

Numerous causative genes have been linked to familial PD^1^,^2^. Of these, mutations in Leucine-rich repeat kinase 2 (*LRRK2*) gene are the most frequent known cause of autosomal dominant PD. *LRRK2* R1628P variant is a risk factor in the Chinese population^3^,^4^.

Epidemiological studies suggest that consumption of caffeine is associated with a reduced risk of PD^5^,^6^. However, risk differs among individuals, as many caffeine takers develop PD and non-caffeine takers are protected from PD. This variability in the degree of neuroprotection conferred by caffeine on different individuals suggests a possible interaction between caffeine consumption and the genetic susceptibility of PD.

Gene environmental interactions studies are rarely examined in PD. Here, we conducted the first case control study to investigate the effect of caffeine intake in subjects with high (*LRRK2* risk variant carrier) and low genetic susceptibility (non carrier) to PD.

## Results

A total of 812 subjects including 378 patients and 434 controls were included in the analysis. The median age of PD patients, median age at onset for PD patients and the median age of controls were 66.0(25–90), 60.0(21–90) and 60.0(21–90) years. In a multivariate logistic regression analysis with adjustment made for the effect of age, gender and family history, we demonstrated that compared to caffeine takers with the wild-type allele (low genetic susceptibility), non-caffeine takers with R1628P variant (high genetic susceptibility) had a 15 times increased risk of developing PD (OR = 15.4, 95% CI = (1.94, 122), P = 0.01), caffeine takers with R1628P (moderate susceptibility) had a 3 times risk (OR = 3.07, 95% CI = (2.02, 4.66), P < 0.001) (Table 1), and non caffeine takers with R1628P was associated with a 2 times risk of PD (OR = 2.18, 95% CI = (1.13, 4.18)). The interaction between the variant and caffeine consumption status was significant with an attributable proportion due to interaction (AP) of about 0.70 (95% CI = (0.32, 1.16)). In the absence of interaction, the OR for the highest-risk group should approximate to 5.86 (calculated based on additive effect of two risk factors).

## Discussion

We examined the interaction between a common *LRRK2* risk variant (R1628P) and caffeine intake in relation to risk of PD. In the multivariate analysis investigating a possible modulating effect of caffeine intake on the *LRRK2* R1628P, the interaction between caffeine intake and R1628P variant on PD risk was statistically significant. Subjects with R1628P variant who did not take caffeine had a 15 times increased risk of PD compared to those who were both carriers of the wildtype and caffeine takers. About 70% of the risk increase was caused by the interaction of R1628P and no caffeine consumption status.

Mutations in the *LRRK2* gene have displayed pleomorphic pathologies that suggest an upstream role of LRRK2 in cellular signaling pathways^2^,^7^. R1628P is located in the COR domain and the substitution of a highly basic polar arginine (R) with a neutral non-proline (P) is likely to cause a conformational change in LRRK2 secondary structure. This change could affect the interaction between the different functional domains of LRRK2 or with other external proteins which might ultimately influence its kinase activity. Dominant missense mutations in *LRRK2* have been postulated to act via a toxic gain of function mechanism coupled with an increase in kinase activity^7^,^8^. However, it isn’t clear if R1628P acts via the kinase dysregulation or other pathways. Whether there is an actual biological interaction between caffeine mediated downstream pathways needs to be further investigated in both *invitro* studies and in animal models.

Our study has some limitations. First, we were unable to evaluate dose response interaction as the information was not fully available. Second, it isn’t clear if our findings could be extrapolated to other populations since gene-environmental interaction may be ethnic specific.

In conclusion, we demonstrated that R1628P variant interacts with caffeine intake in influencing the risk of PD. Caffeine intake would significantly reduce the risk of PD much more in those with high genetic susceptibility compared to those with low genetic susceptibility. Finding environmental factors or lifestyle exposures that modify risk in this genetically susceptible group could shed new light on biological pathways amenable to pharmacological intervention. This could form the basis to explore the possibility of individualized treatment for PD patients with specific genetic background^9^.

## Materials and Methods

Patients diagnosed with PD (based on UK Brain Bank Criteria) by movement disorders neurologists at a tertiary center (received referrals from different outpatient clinics in the country) and controls (healthy volunteers) with no evidence of parkinsonism were recruited. The study subjects were of Chinese ethnicity. All study subjects were evaluated for history of caffeine intake using a validated questionnaire developed in previous studies evaluating gene-environmental factors in PD^10^. Only caffeine intake from coffee and tea was recorded, as coffee and tea (green and black) have previously been shown to be the main source of caffeine intake in our PD population. The study received approval from the Singapore General Hospital/Singhealth institutional ethics committee and subjects gave written informed consent. The methods were carried out in accordance with the approved guidelines.

### Genetic Analysis

Blood samples were collected from all participants for genotyping. DNA was extracted from the blood samples collected using Blood DNA Midi Kit (Qiagen, USA). *LRRK2* c.4883aG > C (R1628P; rs33949390) was genotyped by Taqman SNP genotyping (Life Technologies, Singapore). All positives and selected negatives were confirmed by capillary/Sanger sequencing analysis.

### Statistical Analysis

The effects of R1628P variant, caffeine and combined effects of variant and caffeine on PD were estimated in terms of Odds Ratio (OR) and 95% Confidence Interval (CI) using logistic regression models. Interaction between caffeine and the variant was quantified in terms of attributable proportion due to interaction (AP) together with 95% CI. All statistical analysis was performed using R version 2.15.2 and STATA version 11.0 (Stata Corporation, TX, USA). Statistical significance was defined at P < 0.05.

## Additional Information

**How to cite this article**: Kumar, P. M. *et al.* Differential effect of caffeine intake in subjects with genetic susceptibility to Parkinson’s Disease. *Sci. Rep.* **5**, 15492; doi: 10.1038/srep15492 (2015).

## Figures and Tables

**Table 1:** ORs and APs for developing Parkinson’s disease according to caffeine consumption status and LRRK2 R1628P.

Genotype	caffeineintake	No. of cases/Controls	OR (95% CI)^a^	AP (95% CI)
				0.74 (0.20, 1.29)
GG	Yes	257/369	Reference	
GG	No	81/41	1.91 (0.94, 3.89)	P = 0.07
GC	Yes	18/15	**3.07 (2.02, 4.66)**	P < 0.001
GC	No	10/1	**15.40 (1.94, 122.3)**	P = 0.010

^a^All risk factors in the same logistic regression model and adjusted by age, family history and gender.
